# West Texas Medical Society

**Published:** 1902-12

**Authors:** 


### West Texas Medical Society.

### THE RELATION OF THE MEDICAL PROFESSION TO THE PUBLIC HEALTH.

At the "famous banquet" given by the West Texas Medical Society, at the Menger Hotel, San Antonio, October 23rd, Dr. Frank Paschal, responding to the above toast, said:

Mr. Toastmaster: I appreciate the honor of being called upon to respond to that toast, and shall not be unmindful

> "That long open panegyric drags at best—
> And praise is only praise when well addressed."

But it will be impossible for me to speak on this subject without being a panegyrist.

We know that medicine was born of a necessity, when in the nature of things it was ordained that mankind should die and that in the causation of deaths there should be disease and sickness, hence a sect, if you choose to call our profession such, was created, whose ears have ever been open to hear, and whose hearts are capacitated to heed and respond to the cries of suffering humanity. From the time that medicine broke the shackles that bound it to ignorance and superstition, it has ever been foremost in educating the public how to live that diseases might be prevented. It has brought untold wealth and prosperity to the world; indeed, the North star that illumines the firmament by night, and guides the ocean tossed sailor o'er the pathless deep, is not more fixed, or shines more brightly, than are the purposes and achievements of our profession in the betterment of public health. We need to consider the past to know of the ravages made by zyomotic diseases, when often communities were exterminated by them, is now impossible. The Jews, who followed the teachings of the great sanitarian Moses, were spared to a greater extent than the less careful Christians, and hence were persecuted and accused of poisoning the wells. Not long since ships could not keep the high seas on account of scurvy; today we seldom see a case. Typhus fever that was the scourge of prisons, jails, armies, and of those living under bad sanitary environments, we no longer see—and I dare say that, with the exception of two or three of those who are present, the rest have never seen it. Smallpox that formerly decimated communities is so little known in this country that when it did make its appearance three or four year ago, it was diagnosed as anything from the "Cuban itch" to the "yaws." Cholera, at whose very name the cheeks of the bravest men would blanch and their lips pale, has been brought time and again to the doors of our country and is now no longer dreaded. Today we find that ten years of longevity has been added within the past fifty years. Yellow fever, that upsets commerce and causes a loss of millions of dollars in capital, is now looked upon as being entirely controllable. Improved sanitary methods are diminishing the death rate throughout the civilized world. The future paints to my mind a picture that it would be impossible for the greatest artist to equal on canvas. Not many years hence I see the 25 per cent. of deaths now caused by infants dying under one year of age from preventable diseases reduced to 8 or 10 per cent.; quarantine will become obsolete; contagious diseases, like scarlet fever, measles, and infectious diseases, like diphtheria, will become things of the past; and even "the white plague," so graphically described by Dickens, when he said, "There were times, and often too, when the sunken eye was too bright, the hollow cheek too flushed, the breath too thick and rapid in its course, the frame too feeble and exhausted to escape their regard and notice. There is a dread disease which so prepares its victims, as it were, for death; which so refines it of its grosser aspect, and throws around familiar looks, unearthly indications of the coming change —a dread disease, in which the struggle between soul and body is so gradual, quiet, and solemn, and result so sure, that day by day, and grain by grain, the mortal part wastes and withers away, so that the spirit grows light and sanguine with its lightening load; and feeling immortality at hand, deems it but a new term of mortal life—a disease in which death and life are so strongly blended that death takes the glow and hue of life, and life the gaunt and grisly form of death." Even this I see stamped out. The question naturally arises, how will all of this be done?

Mr. Toastmaster, there was never a battle fought on land or sea to whom the victory wholly belonged to the generals; every man who fought in the battle was entitled to have his brow wreathed with garlands; and so it is with our profession, no man or set of men have accomplished measures which carried alone self-government—that ever attained any degree of success in bettering the public health; but when our profession becomes a unit in advocating measures for the good of the public, we can become "the men behind the guns" and by eliciting the support of the intelligent public we soon see these measures benefiting public health adopted.

In the impetuosity of youth, when not given to looking backwards, but only forwards, I used to think that great measures affecting the public health were slow of enactment; but as I approach "the sere and yellow leaf of age" I realize that all great measures for the weal or woe of communities or nations should be acted upon slowly; we must remember after all that

"We live in deeds, not years; in thoughts, not breaths;
In feelings, not in figures on a dial.
We should count time by heart-throbs; he most lives
Who thinks most, feels the noblest, acts the best.

